# Assessment of Dissolution Profile of Marketed Aceclofenac Formulations

**DOI:** 10.4103/0975-1483.62208

**Published:** 2010

**Authors:** T Soni, N Chotai

**Affiliations:** *Department of Pharmaceutics, Anand Pharmacy College, Sardar Patel University, Anand, Gujarat, India*; 1 *Department of Pharmaceutics, AR College of Pharmacy, Sardar Patel University, Vallabh-Vidyanagar, Gujarat, India*

**Keywords:** Aceclofenac, pair wise procedures, ratio test procedures, Weibull parameters

## Abstract

Statistical comparison of dissolution profiles under a variety of conditions relating to formulation characteristics, lot-to-lot, and brand-to-brand variation attracts interest of pharmaceutical scientist. The objective of this work is to apply several profile comparison approaches to the dissolution data of five-marketed aceclofenac tablet formulations. Model-independent approaches including ANOVA-based procedures, ratio test procedure, and pair wise procedure. The ratio test includes percentage, area under the curve, mean dissolution time, while the pair wise procedure includes difference factor (*f*_1_), similarity factor (*f*_2_), and Rescigno index. In the model-dependent approach, zero order, first order, Hixson-Crowell, Higuchi, and Weibull models were applied to the utilization of fit factors. All the approaches were applicable and useful. ANOVA with multiple comparison tests was found to be sensitive and discriminating for comparing the profiles. Weibull parameters were more sensitive to the difference between two release kinetic data in terms of curve shape and level.

## INTRODUCTION

In 1995, FDA issued a guidance Immediate Release Solid Oral Dosage Forms; Scale-up and Post approval Changes: Chemistry, Manufacturing, and Controls; *in vitro* Dissolution Testing; *in vivo* Bioequivalence Documentation (SUPAC-IR). The *SUPAC-IR* provides recommendations to sponsors of new drug applications (NDA’s), abbreviated new drug applications (ANDA’s), and abbreviated antibiotic applications (AADA’s) who intend, during the post-approval period, to change (i) the components or compositions; (ii) the site of manufacture; (iii) the scale-up/scale-down of manufacture; and/or (iv) the manufacturing (process and equipment) of an immediate release oral formulation. For each type of change, the *SUPAC-IR* also defines (i) levels of changes; (ii) recommended chemistry, manufacturing, and controls tests for each level of change; (iii) *in vitro* dissolution and/or *in vivo* bioequivalence tests for each level of change; and (iv) documentation that should support the change.[1–3]

If dissolution profile similarity is demonstrated for the formulations before and after the changes, then expensive *in vivo* bioequivalence testing can be waived. Various procedures have been proposed for statistical assessment of dissolution profile similarity. These methods include application of either a nested model or an autoregressive time series model to the correlations between cumulative percents dissolved at different time points, and consideration of Mahalanobis distance as a criterion for the assessment of similarity in dissolution profiles between two formulations. Comparison of profiles representing a cumulative event over time is not unique to the pharmaceutical sciences. For equivalence dissolution profile, especially to assure similarity in product performance, regulatory interest is in knowing how similar the two curves are, and to have a measure that is more sensitive to large differences at any particular time point.[4–11]

Aceclofenac is a poorly water-soluble NSAIDS drug according to the BCS system (class II) and its dissolution is rate-limiting step for its absorption.[12–14] Drug absorption from solid dosage forms after oral administration depends on the release of the drug substance from the drug product, the dissolution or solubilization of the drug under physiological conditions, and the permeability across the gastrointestinal tract. Because of the critical nature of the first two of these steps, *in vitro* dissolution may be relevant to the prediction of *in vivo* performance.

In order to evaluate equivalence in dissolution profile among branded and generic formulations of poorly soluble drug, aceclofenac, observations were taken on a given experimental unit over time and Mathematical equations were applied to analyze discrimination in profile and to demonstrate curve shape and level of the profile.

## EXPERIMENTAL DETAILS

### Materials

Aceclofenac (ACE) was gifted from Mepro Pharmaceutical Pvt. Ltd. potassium dihydrogen orthophosphate (Qualigen, Mumbai), sodium bicarbonate (Qualigens, Mumbai) NaOH (Merck) and distill water were used throughout the study. Branded and generic formulations of 100 mg aceclofenac were purchased form a commercial market.

### Methods

#### In vitro dissolution study

Dissolution was performed on five formulations of 100 mg aceclofenac tablets, one branded (Reference) coded S1 formulation and four generic T1, T2, T3, T4 formulations. Dissolution was carried out on six units of each formulation using USP apparatus-II (Paddle) at 37 ± 0.5°C in 900 ml phosphate buffer medium of pH 6.8 at 50 rpm. After appropriate time interval, a sufficient volume of sample was withdrawn and filtered through Whatman filter No. 41. Immediately, same volume of the fresh dissolution medium was transferred to the dissolution flask. Samples were collected at suitable time interval and analyzed spectrophotometrically at 275 nm.

### Statistical evaluation

#### ANOVA-based procedures

One-way ANOVA plus *post hoc* Tukey testing of percentage- dissolved data were applied using Microsoft excel 2007.

#### Model-independent methods

##### Ratio test procedures

Three types of ratio test procedures were performed: Ratio test of percentage dissolved, ratio test of area under the curve, and ratio test of mean dissolution time. Each of these procedures compares the dissolution profile of two formulations at a particular time point. Descriptive statistic form data analysis tool on three types ratio test were performed to analyze standard error and a 90% confidence level for the mean value of ratio of percentage dissolved, AUC, and mean dissolution time.

##### Pairwise procedures

These include difference factor *f*_1_ and similarity factor *f*_2_ (equations 1 and 2) and two indices of rescigno. Rescigno proposed a bioequivalence index (equation 3) to measure the dissimilarity between a reference and a test product-based on plasma concentration as a function of time. This index can also be used for drug dissolution data. Like the ratio test procedure, pairwise procedures compare the dissolution profile of a pair of products and employ a 90% confidence approach. The main advantage of the *f*_1_ and *f*_2_ equations is to provide a simple way to describe the comparison of the data. The *f*_1_ factor measures the percent error between two curves over all the points.

(1) $$f_{1}\;=\;\left[\sum_{i=1}^{\mathrm{in}}\left|R-T\right|\right]/\left[\sum_{i=1}^{p}R\right]\times 100$$

(2) $$f_{2}\;=\;1/\sqrt{50\log\left[1+\left(1/p\right)\sum_{i=1}^{p}{\left(R-T\right)}^{2}\right]}\times 100$$

In both equations, *R* and *T* represent the dissolution measurements at *P* time points of the reference and test, respectively:

(3) $$\mathrm{\xi i}\;=\;{\left\{\frac{\int_{0}^{\infty }\left|d_{R}\left(t\right)-d_{T}\left(t\right){|}^{i}\;\mathrm{dt}\right|}{\int_{0}^{\infty }\left|d_{R}\left(t\right)+d_{T}\left(t\right){|}^{i}\;\mathrm{dt}\right|}\right\}}^{1/i}$$

where *d_R_*(*t*) is the reference product dissolved amount and *d_T_*(*t*) is the test product dissolved amount at each sample time point. i is any positive integer number. This, a dimensional, index always presents values between 0 are 1 inclusive, and measures the differences between two dissolution profiles. This index is 0 when the two release profiles are identical and 1 when the drug from either the test or the reference formulation is not released at all.

#### Model-dependent methods

Model-dependent approaches including zero order, first order, Hixson-Crowell, Higuchi, and Weibull models as described in Table 1 were applied considering amount of drug release from 0 to 90 min. The following plots were made: Cumulative % drug release vs. time (zero order kinetic model); log cumulative of % drug remaining vs. time (first order kinetic model); cumulative % drug release vs. square root of time (Higuchi model), cube root of drug % remaining in matrix vs. time (Hixson Crowell cube root law) and logarithm of the dissolved amount of drug vs. the logarithm of time (Weibull model).[15,16] From the mean ratio of the model parameter and the SE of the mean ratio, a 90% confidence level was assessed.

**Table 1:** Mathematical models used to describe dissolution curves

Zero order	Q_1_ = Q_0_ + K_0_t
First order	l_n_ Q_1_ = l_n_ Q_0_ + Kt
Hixson-crowell	Q_0_^1/3^ – Q_1_^1/3^ = K_s_t
Higuchi	Q_1_ = K_H_^1/2^
Weibull	Log[–ln(12(m))] = blogt–loga

## RESULTS AND DISCUSSION

The FDA suggests some acceptable approaches for establishing similarity of dissolution profiles, such as the model-independent and model-dependent approaches, although any approach would be considered once it had been justified. As a result of the emphasis placed on the comparison of dissolution profile data in FDA guidance, interest among pharmaceutical scientists has focused on methodology used to compare dissolution profile.

Table 2 depicted that the results of application of one-way ANOVA in drug release of aceclofenac-marketed formulations. It was concluded that the differences in the mean values among the treatment groups are greater than would be expected by chance, calculated F-value (3.325) is greater than tabulated F-value (3.055); and there is a statistically significant difference (*P* = 0.038). To evaluate the difference among the four batches, the Tukey test was performed on the results of ANOVA. The results of the Tukey test showed that there was statistically significant difference amongst batch T3 and T4. ANOVA methods takes the variability in the dissolution profile data into account in the comparison at each time point, it ignores the correlation between the dissolution time points.[17]

**Table 2:** Results of one-way ANOVA

ANOVA						
**Source of variation**	**SS**	**df**	**MS**	**F**	***P* - value**	**F crit**

Between groups	6072.356	4	1518.089	3.325194	0.038708	3.055568
Within groups	6848.123	15	456.5415			
Total	12920.48	19				

SS - Sum of squares; MS - Mean square error; df - Degree of freedom

**Figure 1:**
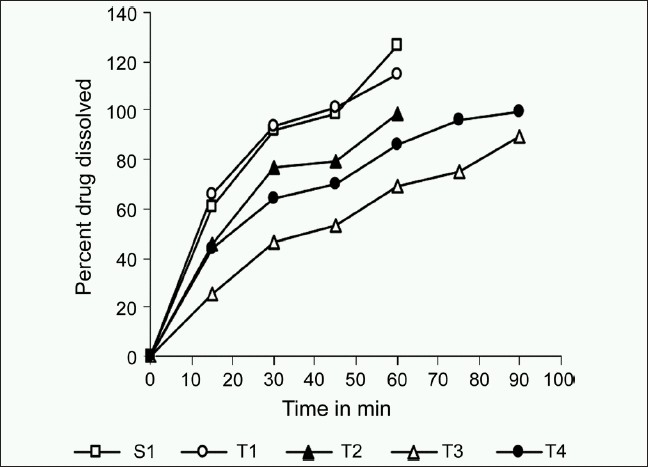
Mean dissolution profile of percentage dissolved

**Figure 2:**
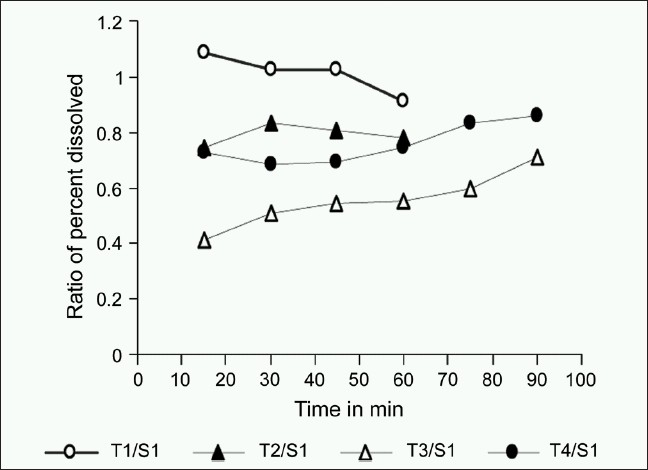
Mean dissolution profile of the ratio of percentage dissolved; [Note: The percent coefficient of variance at the earlier time points should not be more than 20% and at the other time points should not be more than 10%]

**Figure 3:**
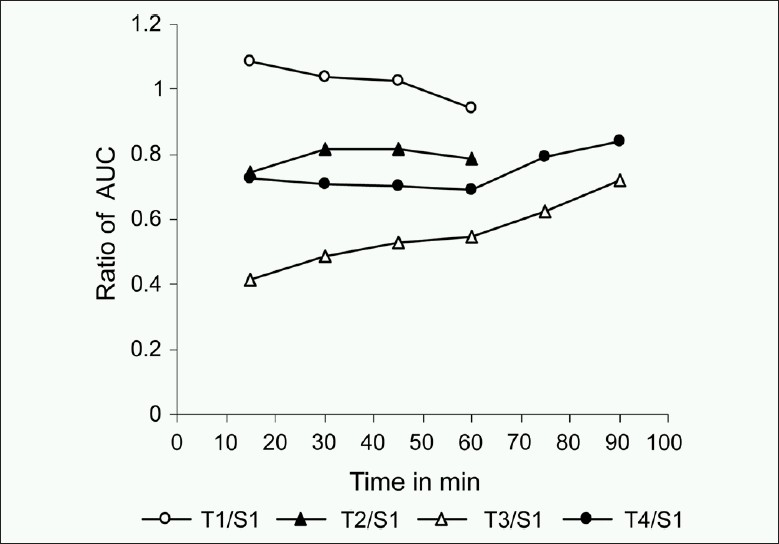
Mean dissolution profile of the ratio of AUC

**Figure 4:**
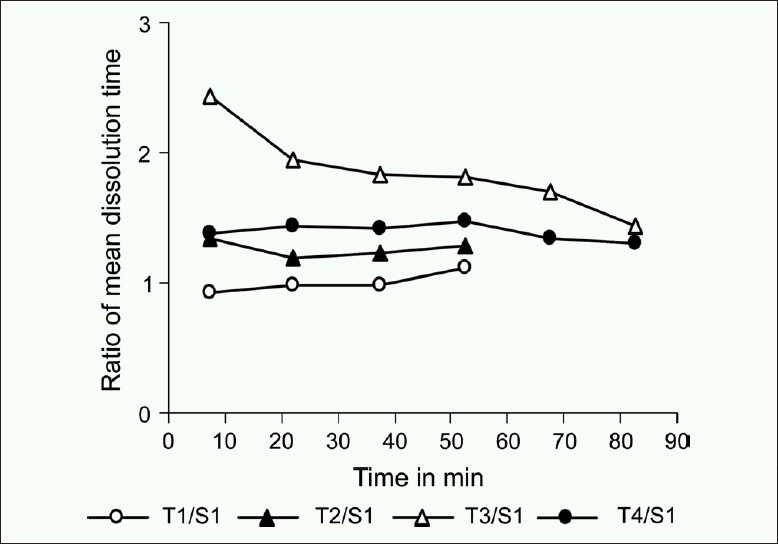
Mean dissolution profile of the ratio of mean dissolution time

Visual graphical interpretations of profiles of the percentage of drug dissolved for formulation S1 and T1-T4 over a 90 min time period in Figure 1 clearly predict similarity in T1 with S1 as compared to T2-T4 formulations. Figures 2–4 represent the ratio of percent dissolved, the ratio of area under the curve of drug dissolved, and ratio of mean dissolution time for test with standard formulation, respectively. Formulations T2 and T4 dissolving greater than half amount of drug (0.75-0.79) to that of reference formulation within 15 min and more than 85% drug were found to be released within 60 min in both the formulations. T3 formulation dissolving less than half amount of drug (0.50-0.59) to that of reference formulation till 60 min and more than 80% drug was found to be dissolved within 90 min. Figure 3 denoted that the ratio of area under the curve for T1 was always within 90% to S1. Over the 60 min, T2 and T4 formulations gave the value of 0.7 and still was not 100% dissolved. For T3, ratio started with 0.4 and reaches the value of 0.7 by 90 min. Table 3 illustrates that throughout the dissolution, the mean ratio of percentage drug dissolved, area under the curve, mean dissolution time for T1 to S1 formulation are always nearer to one and within 90% of that from reference formulation. The ratio of mean dissolution time for T1 was always near to one, while for T2 and T4 it was found to be in a range of 1.2-1.4 times and for T3 it was about 1.8-2.4 times as long as to dissolve as compared to S1. The 90% confidence level for the mean ratio of percentage, AUC, and mean dissolution time were also found to be about twice the standard error (SE).

**Table 3:** Descriptive statistic for the ratio test procedure

Ratio	T1/S1	T2/S1	T3/S1	T4/S1
Percentage				
Mean	1.0124	0.7919	0.5521	0.7568
Std. error	0.0367	0.0188	0.0399	0.0303
90% CL	0.0863	0.0442	0.0804	0.0611
Area under the curve				
Mean	1.0243	0.7903	0.5534	0.7423
Std. error	0.0301	0.0158	0.0444	0.0239
90% CL	0.0708	0.0373	0.0896	0.0481
Mean dissolution time				
Mean	0.9973	1.2587	1.8589	1.3908
Std. error	0.0409	0.0336	0.1339	0.025
90% CL	0.0963	0.0791	0.2698	0.0503

**Table 4:** Mean values of *f*_1_, *f*_2_ and two indices of rescigno

	T1 vs S1	T2 vs S1	T3 vs S1	T4 vs S1
*f*_1_	5.60483	20.49834	48.56718	30.01353
*f*_2_	59.48955	34.95411	16.65016	26.47835
ς_1_	0.028063	0.031224	0.037012	0.032972
ς_2_	0.16752	0.176704	0.192385	0.181583

The *f*_1_ and *f*_2_ equations have been adopted by the FDA in various guidance documents. For the use of mean data, the coefficient of variation at earlier time points should not be more than 20% and not exceed 10% at later time points.[2]

Table 4 depicted the comparison of similarity, dissimilarity index as well as lower and upper rescigno indices. According to the FDA guidance,[12] values of *f*_1_ between zero and 15 and of *f*_2_ between 50 and 100 ensure sameness or equivalence of the two dissolution profiles. Dissolution profile, from point of similarity and dissimilarity criterion, of T1 was best fitted to the S1 formulation. All other formulations were not fitted to the criteria of similarity and dissimilarity. This approach was simple to apply but disadvantage is that both equations do not take into account the variability or correlation structure of the data, are sensitive to the number of points used, and, from a statistical point of view, this method seems to be less discriminating than other methods. The literature revealed several issues relevant to the invariant property of *f*_2_ with respect to the location change, shape of the curve, and the unequal spacing between the sampling time points. The similarity factor is a sample statistic that cannot be used to formulate a statistical hypothesis for the assessment of dissolution similarity. Therefore, it is impossible to evaluate false positive and false negative rates of decisions for the approval of drug products based on *f*_2_. Rescigno proposed a bioequivalence index to measure the dissimilarity between a reference and a test product based on plasma drug concentration time profile. This can also be used on dissolution concentrations. In the present evaluation, lower indices of rescigno were roughly same for all formulation but upper indices values ς_2_ were larger. The indices are more difficult to compute than the *f*_1_ or *f*_2_ equation.[7]

Quantitative interpretation of the values obtained is easier using mathematical equations that describe the release profile in function of some parameters related with the pharmaceutical formulations. The drug transport inside the pharmaceutical system and its release sometimes involve multiple steps provoked by different physical or chemical phenomena, making it difficult, to get a mathematical model describing it in the correct way. Several model-dependent approaches were applied to each dissolution profile. The use of model-dependent methods has been suggested primarily for the situation of many time points. The linearization of ACE dissolution profiles by using the equations presented in Table 5 would characterize the differences found between all batches. Considering the higher determination coefficient (*r*^2^> 0.98), the preferred model that fits best to the dissolution data of reference was the Weibull distribution model. Weibull can describe the dissolution curve in terms of shape and scale parameter. The shape parameter β characterize the curve as exponential (β = 1), S-shaped with upward curvature followed by turning point (β > 1) or parabolic, with a higher initial slope and after that consistent with exponential (β < 1). As dissolution slowed across the formulations, scale parameter became larger and shape factor decreased that indicate that slower formulation possessed lesser sigmoid shape. The lowest value of the ratio of rate constant of test to reference formulation from various model indicate slow release of drug from formulation. The value of β (Shape parameter) in Weibull > 1 reiterates that formulations have a S-shaped profile, but ratio of Weibull β was less than one indicating that test formulations have less S shape as compared to standard. Location parameter (time parameter) Td can be calculated from α and β parameters (α = (T_*d*_)^*b*^) and represents the time interval necessary to dissolve 63.2% of the drug present in pharmaceutical dosage form.[13] In the case of T3 formulation maximum (55 min) was required to dissolve 63.2% drug as compared to other formulation. As compared to reference (16.18 min), faster release was found in T1 formulation (14.28 min).

**Table 5:** Linearization of aceclofenac dissolution profiles using the model-dependent approach (mean value ± SE)

Dissolution models	S1	T1	T2	T3	T4
Zero order K_0_	1.360467 ± 0.054893	1.03235 ± 0.146825	1.09555 ± 0.029064	0.89615 ± 0.012948	0.9514 ± 0.034014
Ratio K_0_ (test/Std)		0.7590	0.805	0.6587	0.6993
R^2^	0.952367 ± 0.007289	0.808917 ± 0.071493	0.906767 ± 0.015782	0.961817 ± 0.018048	0.94215 ± 0.022979
First order K_0_	0.015161 ± 0.000572	0.011669 ± 0.001597	0.015852 ± 0.000288	0.011279 ± 0.00023	0.019115 ± 0.00356
Ratio K_0_ (test/Std)		0.7691	1.0455	0.75	1.2608
R^2^	0.927167 ± 0.00678	0.797233 ± 0.064864	0.8611 ± 0.015516	0.939883 ± 0.024711	0.893767 ± 0.893767
Higuchi K_0_	15.8345 ± 0.629744	12.2008 ± 1.674262	12.91533 ± 0.311756	10.43583 ± 0.15439	11.1286 ± 0.40232
Ratio K_0_ (test/Std)		0.7705	0.8156	0.6590	0.7028
R^2^	0.956517 ± 0.009369	0.835883 ± 0.060702	0.934267 ± 0.014324	0.966083 ± 0.011366	0.954583 ± 0.016311
Hixson Crowell K_0_	–1.10738 ± 0.135942	–1.99378 ± 0.366931	–1.00248 ± 0.19349	–0.23315 ± 0.004429	–0.13922 ± 0.004926
Ratio K_0_ (test/Std)		1.800	0.909	0.211	0.1265
R^2^	0.92855 ± 0.028346	0.856433 ± 0.039812	0.612 ± 0.048742	0.821883 ± 0.016239	0.8913 ± 0.014172
Weibull R^2^	0.9785 ± 0.0045	0.9995 ± 0.0053	0.9163 ± 0.095	0.9721 ± 0.0047	0.9167 ± 0.007
B Shape parameter	1.5221 ± 0.120	1.4079 ± 0.13	0.9197 ± 0.14	1.0626 ± 0.14	1.1243 ± 0.01
Ratio B (test/Std)		0.9249	0.6042	0.6981	0.7386
Td (min) location parameter	16.18279 ± 0.2	14.28369 ± 0. 32	24.24882 ± 0.285	55.09431 ± 0.245	28.48937 ± 0.211
Ratio Td (test/Std)		0.8826	1.4985	3.4045	1.7604
A Scale parameter	0.014444	0.023665	0.053272	0.014122	0.023147

Among other models weibull was considered a good model once it passes the parameters that are sensitive to the change of dissolution profiles. Overall, this study provides experimental evidence for the successful use of the Weibull function in drug release studies.

## CONCLUSION

The main objective of this work was to apply several profile comparison approaches with the intent to investigate several methods and to gain familiarity with the numerical results. It is difficult to assess the extent to which the various approaches described in the literature and FDA guidance are used to compare dissolution profile data. Each method used here for the comparison of dissolution profiles seems to be applicable and useful. It is evident from the literature that no single approach is widely accepted to determine if dissolution profiles are similar. Statistical methods are more discriminative and provide detailed information about dissolution data. Model-dependent methods investigate the mathematical equations that describe the release profile in function of some parameters related to the pharmaceutical dosage forms so the quantitative interpretation of the values is easier. These methods seem to be useful in the formulation-development stage. The *f*_1_ and *f*_2_ are sensitive to the number of dissolution time points and the basis of the criteria for deciding the difference or similarity between dissolution profiles is unclear. The Weibull distribution model has been used for the kinetic analysis of release of aceclofenac formulations.
